# Looking through racism in the nurse–patient relationship from the lens of culturally congruent care: A scoping review

**DOI:** 10.1111/jan.15267

**Published:** 2022-04-20

**Authors:** Mojtaba Vaismoradi, Cathrine Fredriksen Moe, Gøril Ursin, Kari Ingstad

**Affiliations:** ^1^ Faculty of Nursing and Health Sciences Nord University Bodø Norway

**Keywords:** care, culture, literature review, nurse–patient relationship, racism

## Abstract

**Aim:**

This review aimed to identify the nature of racism in the nurse–patient relationship and summarize international research findings about it.

**Design:**

A scoping review of the international literature.

**Data sources:**

The search process encompassed three main online databases of PubMed (including MEDLINE), Scopus and Embase, from 2009 until 2021.

**Review methods:**

The scoping review was informed by the Levac et al.’s framework to map the research phenomenon and summarize current empirical research findings. Also, the review findings were reflected in the three‐dimensional puzzle model of culturally congruent care in the discussion section.

**Results:**

The search process led to retrieving 149 articles, of which 10 studies were entered into data analysis and reporting results. They had variations in the research methodology and the context of the nurse–patient relationship. The thematical analysis of the studies' findings led to the development of three categories as follows: bilateral ignition of racism, hidden and manifest consequences of racism and encountering strategies.

**Conclusion:**

Racism threatens patients' and nurses' dignity in the healthcare system. There is a need to develop a framework of action based on the principles of culturally congruent care to eradicate racism from the nurse–patient relationship in the globalized context of healthcare.

**Impact:**

Racism in the nurse–patient relationship has remained a relatively unexplored area of the nursing literature. It hinders efforts to meet patients' and families' needs and increases their dissatisfaction with nursing care. Also, racism from patients towards nurses causes emotional trauma and enhances job‐related stress among nurses. Further research should be conducted on this culturally variant phenomenon. Also, the participation of patients and nurses should be sought to prohibit racism in healthcare settings.

## 1 INTRODUCTION

According to the American Nurses Association, racism is defined as ‘assaults on the human spirit in the form of biases, prejudices and an ideology of superiority that persistently cause moral suffering and perpetuate injustices and inequities’ (American Nurses Association, 2021) (P.1). Neoliberalism across the globe has made that racism remains invisible in terms of restructuring social classes, producing race categories and racialization (Ahlberg et al., 2019). Racism as prejudice and discrimination based on individuals' race and skin colour is a common healthcare problem across the globe. Ever increasing demographics, globalization and cultural changes in the healthcare context have attracted the attention of policy makers and international authorities to this phenomenon (George et al., 2015).

### 1.1 Background

Racism is the main cause of the patient's harm. Those patients who experience racist discriminations often have poor healthcare outcomes and access to health care, and suffer from mental health issues (Stanley et al., 2019). Racism in its common form as implicit racial bias specially negative attitudes towards the patient of colour can be pervasively observed in the relationship between patients and healthcare providers leading to healthcare disparities (Hall et al., 2015; Sim et al., 2021). It can also hinder appropriate and adequate use of health care, following up screening programmes and preventive behaviours, adherence to the therapeutic regimen and trust in healthcare providers (Powell et al., 2019; Pugh et al., 2021; Rhee et al., 2019). Disparity due to racism leads to the development of new disabilities in patients or even can worsen the present one (Rogers et al., 2015).

Nurses are located at the forefront of patient advocacy and they are expected to address inequities in the provision of care to their clients. However, structural racism can be observed in nursing practice (Iheduru‐Anderson et al., 2021; Villarruel & Broome, 2020). The counter racism role of nurses across healthcare settings emphasizes the identification of discriminatory care, and the development of tolerance, respect and empathy models for other healthcare professionals (Willey et al., 2021).

Ethics is one part of the anti‐racism paradigm with solutions that prohibit racist attitudes and behaviours in health care (Ho, 2016). Racism violates ethical practice among healthcare professions specially among nurses who are committed to the provision of equitable care to patients as the main part of social solidarity (Hamed et al., 2020; Weitzel et al., 2020).

The international research mainly has addressed racism towards patients. The occurrence of racist behaviours from patients towards healthcare professionals should be also investigated to create an equitable environment that hinders racism from both sides. It has been stated that black, Asian and minority ethnic nurses receive a different treatment from patients as being racially stereotyped and are considered less powerful in comparison with white nurses (Brathwaite, 2018; Truitt & Snyder, 2019). Patients' prejudicial and discriminative behaviours in terms of rejecting suggested care, verbal abuse and even physical violence have been described by these nurses as very painful and disrespectful behaviours leading to moral distress and reduction in the quality of patient care (Chandrashekar & Jain, 2020; Keshet & Popper‐Giveon, 2018).

Racism in the healthcare system has a long history. The identification of the nature of racism and its manifestations helps develop appropriate strategies for its elimination from the healthcare system (Mateo & Williams, 2021). The development of actions to tackle racism and racial discriminations has been chosen as a high‐level event at the 76th session of the United Nations General Assembly (UNGA) in 2021 (World Health Organization, 2021). Nevertheless, our knowledge of the extent of racism in healthcare systems and how it can be detected and prevented has remained limited. A probable reason is that racism directly influences the identity and rationality of healthcare professionals, which hinders holding discussions on this phenomenon in public discourses (Hamed et al., 2020). Open discussions on the issue of racism within the nursing profession help identify the underlying causes of racism and eradicate it from the healthcare context (Iheduru‐Anderson et al., 2021).

Further research is needed to better define racial, ethnic and cultural factors contributing to racism in healthcare systems and develop strategies that minimize their impacts on patient care (Godlee, 2020; Paradies et al., 2014).

## 2 THE REVIEW

### 2.1 Aim/s

Previous reviews of the international literature have taken a general perspective towards racism in the multidisciplinary context of health care (Chen et al., 2021; Sim et al., 2021). None of them have investigated racism in the context of the nurse–patient relationship to articulate its characteristics. Therefore, this review of international literature was undertaken to identify the nature of racism in the context of the nurse–patient relationship and summarize international research findings about it.

### 2.2 Design

A scoping review was performed. It is a research method by which the breadth of evidence in a field is mapped and the nature of the research phenomenon is identified (Daudt et al., 2013). The findings of scoping reviews can inform planning for future research and policy making (Westphaln et al., 2021).

This scoping review was carried out based on the review framework suggested by Levac et al. (2010) consisting of the following steps: identification of the research question; literature search and retrieving relevant studies; selection of studies; charting; collating, summarizing data and reporting results; consultation (Levac et al., 2010). These review steps were described under subheadings suggested by the journal's author guidelines.

### 2.3 Search methods

The review question was identified as follows: ‘what is the nature of racism in the nurse–patient relationship?’ The review question was kept broad enough to identify all aspects of this phenomenon in various caring situations, but it focused on related incidents only within the relationship between nurses and patients. It was also formulated by PICO:

P (Population): patients and nurses; I (Interest): racism, and racist attitudes and behaviours in the nurse–patient relationship; Co (Context): all contexts in healthcare including short‐term, long‐term, acute healthcare settings, child, adult, physical and mental healthcare.

The authors designed the review protocol and agreed on its details (Supporting Information 1). They performed a pilot search on Google Scholar and some specialized databases to identify relevant search keywords and phrases. The search process initially was established using the development of keywords, Medical Subject Headings (MeSH), and thesauruses' entry term that were translated into databases. The Boolean method and truncations with the operators of AND/OR were used to create the search sentence, which was pilot‐tested to ensure of its adequacy for retrieving relevant studies and selection of the most relevant databases for conducting the search.

The search sentence included all variants of terms related to nurse, patient, racism (e.g. racial bias, racial prejudice, racial discrimination, covert racism, racial disparity) and relationship (e.g. communication and interaction) in the context of healthcare. After conducting a pilot search, three main online databases that covered the majority of the peer‐reviewed and scientific international literature on racism in the field of nursing consisting of PubMed (including MEDLINE), Scopus and Embase were chosen for conducting the search. A librarian was also consulted to ensure of the accuracy of the search process.

### 2.4 Search outcome

Retrieved studies should have met the following inclusion criteria to be included in the review: original and empirical studies (qualitative/quantitative/mixed methods); focused on the phenomenon of racism; the nurse–patient relationship; racism from patients towards nurses and from nurses towards patients; various healthcare contexts such as short‐term, long‐term, acute healthcare settings, child, adult, physical and mental healthcare; being published in English language in scientific peer‐reviewed journals.

The publication date was restricted as from 1 January 2009 until 31 October 2021 to comprehensively access relevant studies. Any article that did not provide empirical data (e.g. reviews, commentaries, letters, conference proceedings, books) and did not overlap the main domains of the review (i.e. nurse, patient, racism) was excluded. The search coverage was enhanced through conducting a manual search inside some reputed journals with the history of publishing relevant studies and cross‐referencing from selected studies' bibliographies and previous reviews. The EndNote software was used for data management.

### 2.5 Quality appraisal

Risk of Bias assessment and quality appraisal generally are not applicable to scoping reviews. Therefore, all relevant studies were included in the reporting of the review results.

### 2.6 Data abstraction

To prevent bias, the authors (MV, CFM, GU, KI) independently screened the titles and abstracts of retrieved studies. Also, they independently read the full texts of the studies to make decisions on their inclusion or exclusion based on the pre‐determined eligibility criteria. Discussions were held by the authors to reach agreements on the selection of articles and their inclusion in data analysis and reporting results.

An extraction table was used to chart data, facilitate data importing from the selected studies, and categorize their general characteristics based on the author's name, country, publication year, sample and setting and research design.

### 2.7 Synthesis

The analytic framework was developed by drawing tables to collate, summarize and compare the studies' findings in relation to the review phenomenon and present an overview of relevant literature's breadth (Levac et al., 2010). Also, the studies' findings were thematically analysed by comparing their similarities and differences to gain a more abstract and at a higher lever insight into racism in the nurse–patient relationship.

The consultation step is optional and aims at the provision of stakeholders' involvement by suggesting complementary references and giving insights beyond those found in the reviewed literature. The sensitivity of the research topic and the requirement to obtain ethical permissions for collecting data from nurses and patients hindered the authors to follow this step. Therefore, it was removed from the review process.

The review findings were reflected to the three‐dimensional puzzle model of culturally congruent care by Leininger and McFarland (2002) via the main aspects of the cultural competence puzzle at the healthcare provider's and patient's levels consisting of ‘cultural diversity’, ‘cultural awareness’, ‘cultural sensitivity’ and ‘cultural competence’ (Leininger & McFarland, 2002; Schim et al., 2007).

The reason for the selection of culture as the analytical framework in this review lies in its application as the point of reference to the concepts of race and ethnicity. Culture is a dynamic concept, broadly encompasses commonalities and diversities in people and communities, and pervasively influences all aspects of life and healthcare (Schim et al., 2007). This synergy can help heal racism in the healthcare system (Hassen et al., 2021).

The Preferred Reporting Items for Systematic reviews and Meta‐Analyses extension for Scoping Reviews (PRISMA‐ScR) was used to guide this review (Tricco et al., 2018).

## 3 RESULTS

### 3.1 Search results and selection of studies

**FIGURE 1:**
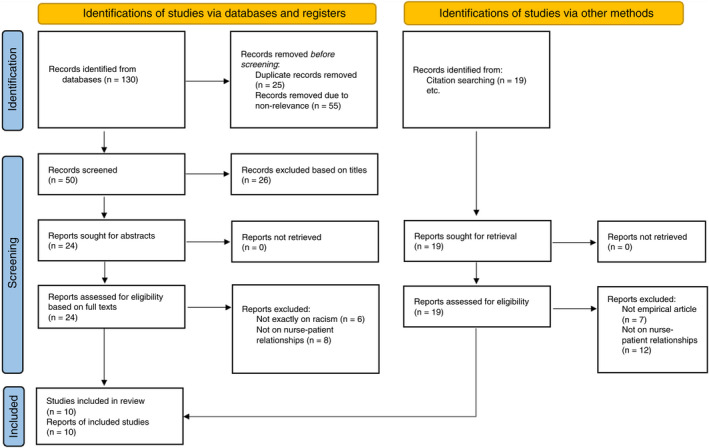
The process of search and inclusion of studies in the scoping review

In the search process, 149 studies were retrieved (Table 1). Duplicates and irrelevant studies were excluded during title screening and abstract reading via holding discussions between the authors. Therefore, 24 articles underwent full‐text reading and assessment, of which 10 studies were entered into data analysis and reporting results given the inclusion criteria (Figure 1).

**TABLE 1:** Results of the different phases of the review

Databases from 2009 to 2021	Total in each database	Records after title reading	Records after abstract reading	Records after full‐text reading
PubMed (including MEDLINE)	13	11	4	3
Scopus	94	32	20	7
Embase	23	6	0	0
Manual search/backtracking references	19	19	0	0
Total of databases	149	68	24	10

### 3.2 Characteristics of selected studies

The general characteristics of the selected studies have been presented in Table 2. They were published between 2009 and 2021 indicating the review coverage for more than one decade. Five studies were conducted in the United States (Benkert et al., 2009; Cottingham et al., 2018; Martin et al., 2016; Purtzer & Thomas, 2019; Wheeler et al., 2014), two in Norway (Debesay et al., 2014, 2021), one each in the United Kingdom (Deacon, 2011), Australia (Lyles et al., 2011) and Canada (McFadden & Erikson, 2020).

**TABLE 2:** List of final articles included in the research synthesis and reporting results

Title of study	Journal	Country	Aim	Methods	Sample and setting
Trust, mistrust, racial identity and patient satisfaction in urban African American primary care patients of nurse practitioners (Benkert et al., 2009)	Journal of nursing scholarship	USA	To analyse relationships between cultural mistrust, medical mistrust, and racial identity and to predict patient satisfaction among African American adults	Descriptive correlational	100 male and female patients, in 3 primary care clinics
How should nurses deal with patients' personal racism? Learning from practice (Deacon, 2011)	Journal of psychiatric and mental health nursing	UK	To promote practice development in the difficult area of managing patients' expressions of personal racism within the clinical environment	Ethnography	Not stated, in one hospital
Provider factors and patient‐reported healthcare discrimination in the Diabetes Study of California (DISTANCE) (Lyles et al., 2011)	Patient education and counselling	Australia	To examine healthcare provider's level factors and reported discrimination in the healthcare setting	Survey	12,151 patients from the metropolitan area based on data registry
The experience of discrimination by US and Internationally educated nurses in hospital practice in the USA: a qualitative study (Wheeler et al., 2014)	Journal of advanced nursing	USA	To identify nurses' interactions with patients and their families and other healthcare personnel, and strategies for managing interactions and rationales behind their selected strategy	Qualitative using structuration theory	42 internationally educated and 40 USA‐educated nurses in 2 hospitals
Facing diversity under institutional constraints: challenging situations for community nurses when providing care to ethnic minority patients (Debesay et al., 2014)	Journal of advanced nursing	Norway	To explore challenges faced by community nurses when providing home healthcare to ethnic minority patients	Qualitative using hermeneutic	19 nurses in 4 home care city districts
Racial differences in parental satisfaction with neonatal intensive care unit nursing care (Martin et al., 2016)	Journal of perinatology	USA	To explore satisfaction and expectations about nursing care between racial groups in the neonatal intensive care unit	Qualitative grounded theory	249 families in 30 children hospitals
‘I Can Never Be Too Comfortable’: race, gender and emotion at the hospital bedside (Cottingham et al., 2018)	Qualitative health research	USA	To examine how race and gender shape nurses' emotional practice	Abductive approach	48 nurses working in healthcare settings in 2 cities
Intentionality in reducing health disparities: Caring as connection (Purtzer & Thomas, 2019)	Public health nursing	USA	To examine healthcare disparities within the context of patient–nurse relationships	Descriptive qualitative	11 nurses in 1 rural county
How nurses come to race: racialization in public health breastfeeding promotion (McFadden & Erikson, 2020)	Advances in nursing science	Canada	To understand how race becomes ascribed through nursing theory and day‐to‐day workplace socialization processes	Ethnography	30 nurses in 6 public health units
Healthcare professionals' encounters with ethnic minority patients: The critical incident approach (Debesay et al., 2021)	Nursing inquiry	Norway	To explore healthcare professionals' experiences of working with ethnic minority patients by using the critical incident technique	Focus group on critical incident cases	6 public health nurses in 1 hospital

The studies mainly used the qualitative design (Cottingham et al., 2018; Deacon, 2011; Debesay et al., 2014, 2021; Martin et al., 2016; McFadden & Erikson, 2020; Purtzer & Thomas, 2019; Wheeler et al., 2014), but two studies used the quantitative design (Benkert et al., 2009; Lyles et al., 2011). They were conducted in hospitals and community healthcare settings and were categorised based on their focuses as follows: patients' trust in nurses in relation to their racial identities (Benkert et al., 2009), nurses' confrontation with the racist expression by patients (Deacon, 2011), patient‐reported racial discrimination and communication with nurses (Lyles et al., 2011), perspectives of internationally educated nurses and American educated nurses about interactions with patients (Wheeler et al., 2014); nurses' experiences of home care provision to ethnic minority patients (Debesay et al., 2014); satisfaction of various racial groups of parents with neonatal nursing care (Martin et al., 2016); experiences of nurses of colour about negotiating with patients (Cottingham et al., 2018); health disparities during the nurse–patient relationship (Purtzer & Thomas, 2019); racializing mothers and breastfeeding (McFadden & Erikson, 2020); nurses' critical encounters with ethnic minority patients (Debesay et al., 2021).

### 3.3 Racism in the nurse–patient relationship

The thematic analysis of the studies' findings led to the development of three categories: bilateral ignition of racism, hidden and manifest consequences of racism and encountering strategies.

#### 3.3.1 Bilateral ignition of racism

Pervasive racist perspectives and stereotypies among patients and nurses shaped their racist behaviours and negatively impacted the nurse–patient relationship.

Implicit bias among nurses towards racial and ethnic minorities was available in the form of having a general assumption about minorities, and was reflected through indirect negative racist expressions during the nurse–patient communication rather than direct impolite racist remarks (Debesay et al., 2021). From the macro‐perspective, power bias innated in the patient–nurse relationship. Considering patients in a weaker social position was the main cause of racist incidents (Debesay et al., 2021; Purtzer & Thomas, 2019).

Racism in institutional practice and policies also contributed to negative stereotypes. Ethnic minority patients who did not follow institutional guidelines were considered outsiders. They were labelled as clients who could not get integrated into social values, and were subject to racist remarks. Education in terms of hidden curricula and by learning from others when nurses worked in healthcare settings established racist stereotypes and attitudes towards patients (McFadden & Erikson, 2020; Purtzer & Thomas, 2019).

From a micro‐perspective, bias and stereotypical attitudes leading to racist behaviours rooted in nurses' personal perspectives towards ethnic minorities who had limited language proficiencies and substance dependencies, and suffered from mental illness (Debesay et al., 2021; Lyles et al., 2011; McFadden & Erikson, 2020; Purtzer & Thomas, 2019). Instead of assessing patients' cultural and ethnic backgrounds and investigating their cultural identities, nurses guessed on patients' cultural characteristics and needs based on their habits and last names to plan healthcare interventions (McFadden & Erikson, 2020). Failure to follow‐up nurses' health‐related advice, patients' socio‐economic factors and stereotypical attitudes developed by patients themselves towards their own physical and mental in‐capabilities enhanced racial distortions among nurses (McFadden & Erikson, 2020; Purtzer & Thomas, 2019).

On the other hand, patients' racism towards nurses was revealed in the experiences of nurses. Patients committed racial aggression when nurses were unable to meet their needs, which in some cases were unreasonable and beyond the defined nurse–patient relationship. Also, nurses' communication with accent triggered racist behaviours in patients (Cottingham et al., 2018; Debesay et al., 2021; Wheeler et al., 2014).

Nurses did not consider their position higher than patients in the nurse–patient interaction rounds. Nevertheless, some patients placed nurses at the lowest hierarchy of humanistic relationships and labelled them as subordinates. Patients' perspectives of nurses' ethnicity and cultural backgrounds determined the levels of nurses' competencies to provide care and receive respect. Nursing care was rejected by some patients, because of their personal orientations towards nurses' culture and ethnicity (Cottingham et al., 2018; Wheeler et al., 2014).

#### 3.3.2 Hidden and manifest consequences of racism

Negative consequences of racism in the nurse–patient relationship were reported by both patients and nurses. Working in an environment in which stereotypical and racist attitudes influenced the nurse–patient relationship triggered the feeling of insecurity and uncertainty. Fear of making mistakes and crossing ethnic and cultural boundaries of minorities and the possibility of conflicts between patients' and families' beliefs, and nursing interventions enhanced work‐related stress among nurses (Debesay et al., 2014, 2021). Nurses faced uncertainties with regard to how withhold their own personal prejudices and at the same time provide nursing care according to professional commitments (Debesay et al., 2021; Purtzer & Thomas, 2019).

Apparently, health disparities occurred given tensions between nurses and patients rooted in racist perspectives. They hindered nurses' efforts to provide appropriate care to patients and improve the nurse–patient relationship. When patients were not given opportunities to assert their cultural identities, they were discouraged to follow nurses' interventions and health‐related advice leading to more healthcare issues (Debesay et al., 2021; McFadden & Erikson, 2020; Purtzer & Thomas, 2019).

Patients mainly were dissatisfied with receiving support by nurses and complained about nurses' superior, cold and without sympathy communication style, inattention to their caring needs, not receiving suitable education, not spending enough time with patients and frequent nurses' turnover (Lyles et al., 2011; Martin et al., 2016; McFadden & Erikson, 2020).

A negative consequence of nurses' racist behaviours was the development of a negative perspective among patients towards the healthcare system. Racism was generalized to the whole healthcare system rather than taking it as a personal matter in the nurse–patient relationship (Benkert et al., 2009). Consequently, patients displayed disappointment or anger to all nurses and retaliated it, which damaged the sense of justice and pride even among those nurses who did their best to provide equitable care to patients (Debesay et al., 2021).

Those nurses who were subject to racist behaviours from patients experienced job‐related and emotional stress, which depleted their psychological resources and energy to deliver care. Assumption of incompetence due to racism led to emotional shift and encouraged nurses to retaliate. Therefore, instead of concentration on patient care, nurses focused on managing frustration and emotional trauma (Cottingham et al., 2018). Social isolation and disconnection, and leaving the nursing profession were some risky consequences of racist incidents (Cottingham et al., 2018; Wheeler et al., 2014).

#### 3.3.3 Encountering strategies

Nurses and patients used strategies to avoid racism or at least minimize its impact on the nurse–patient relationship. Respect, trust and active participation in nursing care worked quite fine against stereotypical and racist behaviours. Compassionate care and respectful style of communication by nurses, friendliness, patience and taking care of patients' concerns and spending enough time for education were highlighted. These strategies could be all summarized into being patient‐centred (Lyles et al., 2011; Martin et al., 2016; Purtzer & Thomas, 2019).

Cultural mistrust as the outcome of racism could be avoided through the development of racial concordance in the nurse–patient relationship. The suggested strategy to achieve concordance was the provision of care by those nurses who had similar cultural and racial backgrounds to those of patients (Benkert et al., 2009; Lyles et al., 2011). Also, cultural understanding through the acknowledgment of patients' culture and learning about their values, ceremonies and traditions, integration of patients' values into nursing care and setting healthcare goals to preserve patients' cultural identity was required. Avoiding the creation of an unpleasant atmosphere in the nurse–patient relationship through not directly questioning patients' cultural characteristics, and balancing between care delivery and cultural rituals such as touching patients and undressing them helped prevent crossing cultural borders and creating the feeling of racism. Moreover, leading ethnic minority patients in healthcare journey and covering the gap between them and the requirements of the healthcare system were the main strategies for patient advocacy (Debesay et al., 2014, 2021; McFadden & Erikson, 2020; Purtzer & Thomas, 2019).

When nurses faced racism from patients, they tried to avoid personalizing racist incidents and made jokes of them to control their anger and defend their own emotional well‐being. They considered such sorts of abuses one part of their daily work life that should be coped with (Cottingham et al., 2018; Deacon, 2011). Given the lack of policies in healthcare settings to manage racism, nurses coped with the situation and rationalized racist behaviours to reduce related emotional burdens. They tried to ignore racism and attributed it to patients' background diseases, age, previous negative life experiences and inability to take the responsibility of their own behaviours. As a confrontation strategy, some nurses reported the racist incident to authorities, used medications to calm patients, applied distraction techniques to patients, asked patients to refrain from being assaultive, and informed patients of their behaviours. In the worst case, some nurses decided to change their workplace (Cottingham et al., 2018; Deacon, 2011; Wheeler et al., 2014).

## 4 DISCUSSION

This scoping review of the international literature aimed to identify the nature of racism in the nurse–patient relationship and summarize international research findings about it. An overview of the breadth of the international literature on this phenomenon was presented consisting of three categories developed by the authors. Culture is intertwined with the phenomenon of racism and can critically influence the nurse–patient relationship (Crampton et al., 2003). Racism as an individual and systemic prejudice is imprinted in cultural artefacts and discourses (Salter et al., 2017). Racist perspectives, stereotypies and behaviours from patients and nurses can be attributed to cultural bias as the interpretation of situations and others' actions according to own set of personal perspectives, experiences and cultural standards.

**FIGURE 2:**
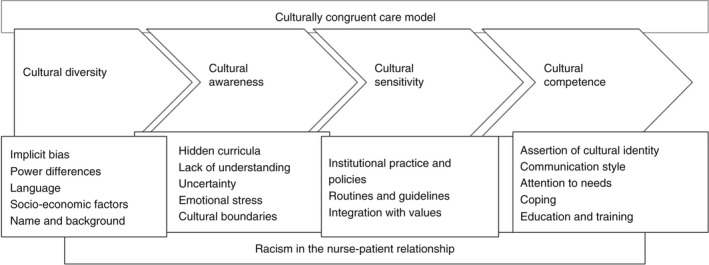
The review findings in connection to the culturally congruent care model

Delivering unbiased and individualized care to culturally diverse patients is influenced by nurses' cultural competencies. Also, patients' personal attitudes and perspectives, and balancing the power between nurses and patients in healthcare situations are crucial to the development of an appropriate climate for patient care (Oxelmark et al., 2018; Vaismoradi et al., 2015). Accordingly, the findings of this review were discussed using the main aspects of the cultural competence puzzle consisting of cultural diversity, cultural awareness, cultural sensitivity and cultural competence as the elements of the three‐dimensional puzzle model of culturally congruent care at nurses' and patients' levels (Leininger & McFarland, 2002; Schim et al., 2007). The dimensions of this model have also the capacity to be the part of the patient's participation in the provision of culturally congruent care (Schim et al., 2007). Therefore, our discussion using this model covers racist behaviours from both patients and nurses. A summary of the review findings in connection to the culturally congruent care model has been presented in Figure 2.

### 4.1 Cultural diversity

According to the review findings, racist behaviours in the nurse–patient relationship rooted in implicit and power biases demonstrating that the patient and the nurse had a low social position. Stereotypical attitudes were developed towards patients with limited language proficiencies, low socio‐economic conditions and different last names and cultural backgrounds.

Globalization is the cause of cultural diversities in health care. Similarities and differences between cultures in terms of race, ethnicity, nationality and ideology shape humanistic relationships in health care (Schim et al., 2007). The patient's and healthcare provider's cultural contexts are crucial in the development of the therapeutic relationship. The establishment of a constructive relationship between nurses and patients without the acknowledgment of their cultural diversities is impossible (Gopalkrishnan, 2018). Marginalization of ethnicities and minorities in the healthcare system should be avoided and instead their cultural diversities should be acknowledged and respected. All measures should be taken to avoid tensions when contacts between cultures occur. The assessment of cultural diversities is the cornerstone of planning for the provision of culturally congruent care through appropriate exposure to cultural differences and prevention of racism (Schim et al., 2007). Diversities in nurses' cultural backgrounds have been shown to be advantageous for the healthcare system in terms of improving the quality of patient care and healthcare economy (Gomez & Bernet, 2019).

### 4.2 Cultural awareness

In this review, education had an influence on the development of racism towards patients through hidden curricula and by learning at the workplace. Nurses felt uncertain about how to provide care that was congruent to patients' cultural backgrounds without having stress about crossing patients' cultural boundaries. Those nurses who faced racism from patients often were unable to manage the situation, were emotionally overloaded, and lost their concetration on the provision of care. Similarly, patients' racist behaviours towards nurses were attributed to a lack of understanding of nurses' cultural backgrounds.

Gaining knowledge about and recognition of other cultures help to identify the uniqueness of each culture and commonalities between the cultures. Cultural awareness aims at identifying similarities and differences between cultures in terms of religious rituals, routines, preferences and behaviours. It recognizes interpersonal comfort zones and customizes care to them, and suggest a method by which people can interact with others' cultures in the caring relationship leading to the delivery of culturally sensitive care (Schim et al., 2007).

Cultural awareness often happens in the process of informal nursing education because direct education may not be able to provide sufficient opportunities for nurses to become culturally aware (Hultsjö et al., 2019; Kaihlanen et al., 2019). Raising awareness about caring situations in which misinterpretations may occur help with the detection of underlying causes and finding a counteraction framework by which an equitable communication is made with patients and their satisfaction is preserved (Crawford et al., 2017; Tan & Li, 2016). Comparisons of cultures and discovery of common ethical values in the nursing profession help develop skills for the creation of dialogue between individuals' and facilitate integration to the global nursing context (Leung et al., 2020).

### 4.3 Cultural sensitivity

In this review, institutional practice and policies contributed to the development of negative stereotypes by which ethnic minority patients who did not follow institutional guidelines were subject to racist behaviours. Cultural sensitivity consists of individuals' attitudes towards others and themselves and understanding others' cultural characteristics. It motivates individuals to be cross‐cultural and acknowledges others' cultural heritages. Judging others' cultures based on own culture is against the principle of cultural sensitivity (Schim et al., 2007). Measures taken in healthcare systems to reduce bias and racism should encompass all types of inequalities among ethnic minorities (Sim et al., 2021). Improving cultural sensitivity enhances cultural intelligence and facilitates understanding the impact of culture on health and diseases. Therefore, the provision of intercultural healthcare based on understanding differences and similarities between cultures leads to the reduction of health inequalities and improvement of healthcare quality (Göl & Erkin, 2019; Yilmaz et al., 2017). Public and social media have important roles to tackle the problem of patients' racist behaviours towards nurses in care situations. They can debate healthcare policies, promote public health behaviours, educate patients and inform them of cultural norms in healthcare settings and engage them in the development of an environment that respects cultural diversities. Improving cultural sensitivity involves an increased focus on human rights. Individuals' equal worth and rights regardless of race, ethnicity, language and religion lay the foundation of human rights (United Nations, 2021).

### 4.4 Cultural competence

The findings of this review showed that patients could not assert their cultural identities and were dissatisfied with nurses' inappropriate communication style and lack of attention to their needs. Although there was no indication of training to nurses about culturally congruent care in our findings, the main focus of strategies to avoid racism by nurses was to acknowledge the patient culture, behave respectfully and provide compassionate care. This coping strategy supported nurses' personal well‐being and at the same time prevented the creation of negative stereotypes towards patients' cultural backgrounds. It is the demonstration of a series of behaviours and taking related actions indicating that healthcare professionals know how to acknowledge cultural diversities and are aware of and sensitive towards the patient's culture (Schim et al., 2007).

In the context of health care, it is to adapt care and comply skills to patients' needs. Being culturally competent facilitates culturally congruence care in the nurse–patient relationship. Cultural competence for ethnic minorities requires organizational support (Taylor, 2005) and it should include work at the system level (Sharifi et al., 2019). Cultural education and training have been emphasized as mitigating strategies that can reduce racism and bias, and enhance cope with cultural diversities. Cultural competence is an important strategy by which health inequities can be addressed (Horvat et al., 2014). It requires practising self‐reflexivity on routines that cause racism and bringing implicit bias to own conscious (Bradby et al., 2021; Medlock et al., 2017; Olukotun et al., 2018). Training about diversities and being exposed to cultural differences in practical placements can promote cultural competence and consequently interaction with culturally diverse patients (Levey, 2020; McLennon et al., 2019). Promoting cultural competence among healthcare providers prevents healthcare encounters and reduces shame and embarrassment among care receivers (Flynn et al., 2020).

### 4.5 Limitations and suggestions for future studies

This scoping review provided an overview of international knowledge about racism in the nurse–patient relationship in spite of retrieving a few empirical studies on this important phenomenon. More studies might have been published in languages other than English that could not be included in this review, and should be considered by future researchers. Racism in the nurse–patient relationship has remained a less explored area of nursing research specially regarding racism from patients towards nurses. Therefore, more studies about racism in the context of the nurse–patient relationship and in various healthcare contexts should be conducted to improve our knowledge of this culturally variant phenomenon and devise a general unified strategy for the eradication of racism from the nurse–patient relationship.

## 5 CONCLUSION

Racism threatens patients' and nurses' dignity in the healthcare system. It hinders efforts to meet patients' and families' needs and increases their dissatisfaction with nursing care leading to the loss of trust in nurses and reduction of quality of care. Also, racism from patients towards nurses causes emotional trauma and enhances job‐related stress among nurses leading to their turnover. Nurses often apply coping strategies to relieve the emotional pressure of racist incidents and protect their own emotional well‐being.

Racism in the globalized context of healthcare should be prevented and nurses' and patients' well‐being and dignity should be preserved. It needs the establishment of acts and legislations that prohibit racist behaviours and enforce their report to healthcare authorities to seek support and prosecute racist people. Also, there is a need to develop a framework of action based on the principles of culturally congruent care to eradicate racism from the nurse–patient relationship in the globalized context of healthcare.

The practical implications of the review findings based on the culturally congruent care model are as follows:

- Development of a practical guideline to help nurses and patients acknowledge cultural diversities and promote their awareness and sensitivity towards other cultures;
- Improvement of nurses' cultural competence through education and training about how to avoid racism during the provision of care to patients with different cultural backgrounds;
- Development of policies and practical strategies to ensure that patients are held responsible for racist behaviours that create a toxic environment for healthcare professionals;
- Comparing cultures and removing misperceptions about other cultures through communication and dialogue between nurses and patients;
- Rectification of institutional policies contributing to the creation of stereotypies about cultural minorities;
- Use of public and social media to inform patients of cultural norms in healthcare settings;
- Collaboration between associations supporting the human rights of nurses and patients for the development and implementation of zero‐tolerance and anti‐racism policies;
- Emphasis on the equal worth of people and human rights in healthcare settings;
- Cultural socialization of nurses through education and training about customizing care to patients' cultural backgrounds, demonstrating respect and providing compassionate care;
- Active screening and detection of stereotypes, implicit bias and racist attitudes among nurses through self‐reflexivity.

## CONFLICT OF INTEREST

The author(s) declared no potential conflicts of interest with respect to the research, authorship, and/or publication of this article.

## AUTHOR CONTRIBUTIONS

MV was involved in review design. MV, CFM, GU and KI were involved in data acquisition, analysis and interpretation for important intellectual content, drafting the manuscript and revising it for intellectual content and giving final approval of the version to be published in the journal.

### PEER REVIEW

The peer review history for this article is available at https://publons.com/publon/10.1111/jan.15267.

## Supporting information


Data S1.

Data S2.

## Data Availability

Authors do not want to share the data.
