# Supplementary material for: Looking through racism in the nurse–patient relationship from the lens of culturally congruent care: A scoping review
Source: J Adv Nurs. 2022 Apr 20;78(9):2665–77. doi: 10.1111/jan.15267 (PMC9544978; doi:10.1111/jan.15267)
Supplement: Supplementary file 1 — Data S1. [file JAN-78-2665-s001.docx]

**Supplementary file.**

Table 1. The protocol of the scoping review of racism in the nurse-patient relationship.

| **Phase** | **Description** |
| --- | --- |
| **Review question** | What can be found in the international literature regarding racism in the nurse-patient relationship?  Branching questions to the review topic are:  What are the main aspects of racism in the nurse-patient relationship?  What factors cause racism and racist behaviours in the nurse-patient relationship?  How can racism in the nurse-patient relationship be prevented?  What is the gap of knowledge about this topic in the international literature? |
| **Review type** | Scoping review of the international literature. |
| **Language** | English |
| **Study designs** | All original research-based studies such as qualitative, quantitative, and mixed-methods studies will be included. Reviews, commentaries, letters, case reports, case studies, and books will be excluded. |
| **PICO statement** | P (Population): patients and nurses; I (Interest): racism, and racist attitudes and behaviours in the nurse-patient relationship; Co (Context): all contexts in healthcare including short-term, long-term, acute healthcare settings, child, adult, physical and mental health care. |
| **Literature search** | A librarian assists with the search process.  Electronic databases such as PubMed [including MEDLINE], Scopus, Embase will be searched for retrieving studies published from 2009 to the time of our review.  Manual search and grey literature: Important journals for manual search consisting of journal of transcultural nursing, health affairs, and [journal of racial and ethnic health disparities](https://www.springer.com/journal/40615/) will be identified and searched. It also includes cross-references from bibliographies for improving the search coverage.  Keywords: After a pilot search on databases, the combination of keywords for search to be used via the Boolean Method are as follows:  nurs* AND (patient OR client) AND (racism OR racist OR “racial bias” OR “racial prejudice” OR “racial discrimination” OR “racist behaviour” OR “racial disparity” OR “covert racism” OR “implicit racism”) AND (“nurse-patient relationship” OR “nurse-patient relationships” OR “nurse-patient communication” OR “nurse-patient interaction”) |
| **Literature selection** | Selecting original studies based on titles, abstracts and full-text of articles.  Pre-tested inclusion criteria for original and scientific content are as follows: studies with qualitative, quantitative and mixed methods designs; explicit interest to racism; racism from patients towards nurses and from nurses towards patients; in the nurse-patient relationship; in short-term and long-term healthcare settings as well as community healthcare settings; published in English language, and in peer-reviewed scientific journals.  Articles without an exact relevance to the nurse-patient relationship or with a concentration on other healthcare professionals will be excluded. |
| **Research synthesis** | For data extraction, a table will be drawn comprising the following sections: the first author’s surname, publication year, and the country where the study was conducted; study design, sample size, and setting; data relating to racism in any form in the nurse-patient relationship. The accuracy of gathered data before the research synthesis will be assessed through double checking by the authors.  The Levac et al.’s methodological framework consisting of the following steps: identifying the research question, identifying relevant studies, study selection, charting the data, collating, summarizing, and reporting results, and consultation. The last step as consultation is irrelevant to our review context.  Levac D, Colquhoun H, O'Brien KK. Scoping studies: advancing the methodology. Implement Sci. 2010 Sep 20;5:69. doi: 10.1186/1748-5908-5-69. PMID: 20854677; PMCID: PMC2954944. |
| **Equator guideline** | The process of reporting the review will be guided by the methodological checklist of the extension of PRISMA for scoping reviews.  Tricco, A. C., Lillie, E., Zarin, W., O'Brien, K. K., Colquhoun, H., Levac, D., ... & Straus, S. E. (2018). PRISMA extension for scoping reviews (PRISMA-ScR): checklist and explanation. Annals of internal medicine, 169(7), 467-473. |
| **Funding sources/sponsors** | This research has received no external funding. |
| **Conflicts of interest** | The authors declare that the research is conducted in the absence of any commercial or financial relationships that could be construed as a potential conflict of interest. |
